# False Alarm Reduction in BSN-Based Cardiac Monitoring Using Signal Quality and Activity Type Information

**DOI:** 10.3390/s150203952

**Published:** 2015-02-09

**Authors:** Tanatorn Tanantong, Ekawit Nantajeewarawat, Surapa Thiemjarus

**Affiliations:** 1 School of Information, Computer, and Communication Technology, Sirindhorn International Institute of Technology, Thammasat University, Pathum Thani 12000, Thailand; E-Mail: tanatorn@siit.tu.ac.th; 2 National Electronics and Computer Technology Center, Pathum Thani 12120, Thailand; E-Mail: surapa.thiemjarus@nectec.or.th

**Keywords:** false alarm reduction, arrhythmia classification, signal quality classification, activity classification, body sensor network, machine learning, rule-based expert system

## Abstract

False alarms in cardiac monitoring affect the quality of medical care, impacting on both patients and healthcare providers. In continuous cardiac monitoring using wireless Body Sensor Networks (BSNs), the quality of ECG signals can be deteriorated owing to several factors, e.g., noises, low battery power, and network transmission problems, often resulting in high false alarm rates. In addition, body movements occurring from activities of daily living (ADLs) can also create false alarms. This paper presents a two-phase framework for false arrhythmia alarm reduction in continuous cardiac monitoring, using signals from an ECG sensor and a 3D accelerometer. In the first phase, classification models constructed using machine learning algorithms are used for labeling input signals. ECG signals are labeled with heartbeat types and signal quality levels, while 3D acceleration signals are labeled with ADL types. In the second phase, a rule-based expert system is used for combining classification results in order to determine whether arrhythmia alarms should be accepted or suppressed. The proposed framework was validated on datasets acquired using BSNs and the MIT-BIH arrhythmia database. For the BSN dataset, acceleration and ECG signals were collected from 10 young and 10 elderly subjects while they were performing ADLs. The framework reduced the false alarm rate from 9.58% to 1.43% in our experimental study, showing that it can potentially assist physicians in diagnosing a vast amount of data acquired from wireless sensors and enhance the performance of continuous cardiac monitoring.

## 1. Introduction

Home health monitoring has gained a rapid surge of interests for observing deviations in health status from the norm in early phases and automatically alerting paramedics or physicians. According to a report from the World Health Federation [1], over 70 percent of all cardiac and breathing emergencies occur at home. In order to provide patients with continuous cardiac healthcare services, new techniques such as wireless sensors [2–4] and real-time automated diagnosis of Electrocardiogram (ECG) [5–10] should be integrated into a traditional cardiac monitoring system. Some real-time cardiac monitoring systems with wireless sensors have been proposed [11–14], with their potential values being reported. However, ECG signals recorded using wireless Body Sensor Networks (BSNs) during activities of daily living (ADLs) are not only often interrupted by noises generated from wireless monitoring devices, but also easily contaminated with noises arising from body movements [15–17]. These factors can lead to high false alarm rates in continuous monitoring [18–20]. Further investigations on the effects of signal quality and ADLs are thus required.

Several techniques have been proposed for real-time wireless ECG continuous monitoring. In [11], Oresko *et al.* developed a smartphone-based wearable platform for real-time cardiovascular disease detection. A relatively small selected portion of data (5421 beats) from the MIT-BIH arrhythmia database (with a total of 109,935 beats) [21] was used for classifying normal beats and four types of arrhythmia beats. In [12], Lin *et al.* proposed a wireless, ambulatory, real-time, and auto alarm intelligent telecardiology system for improving cardiac healthcare services. A lightweight and power-saving wireless ECG device, equipped with a built-in automatic warning expert system, was developed. In their data collection, 10 normal subjects and 20 subjects with atrial fibrillation were asked to maintain regular breathing and abstain from body movement and speaking during 5 min of data recording. Winkler *et al.* [13] developed a system for remote monitoring of chronic failure patients using mobile phone networks. ECG signals were acquired from 30 healthy volunteers for 26 days, and only 6% of the obtained ECG recordings were reported to have sufficient diagnostic quality for rhythm analysis and single beat measurement. In [14], Andreoli *et al.* proposed a framework based on wireless BSNs for supporting continuous cardiac monitoring. ECG signals were collected from subjects while they were performing ADLs such as sleeping, watching TV, and walking. Using a threshold-based algorithm, time-domain features extracted from RR intervals were used for identifying emotional stress levels of the monitored subjects. Noises arising from body movements and the effects of signal quality on the classification results were not yet considered in these studies.

ECG signal quality assessment has attracted a rapid surge of research interests in recent years. The Physionet/Computing in Cardiology Challenge 2011 dataset (CinC dataset) [22], consisting of 2000 12-lead ECG recordings, each 10 s long, has been widely used for training and testing quality assessment models. In [23], Hayn *et al.* investigated the use of ECG quality measures for quality assessment of ECG signals using data from the CinC dataset with a threshold-based algorithm. Their simplified algorithm for Android platforms was the winning entry in event 3 of the 2011 Computing in Cardiology Challenge. In [24], Clifford *et al.* developed an automated algorithm to detect poor-quality ECG signals. Features reflecting morphological, statistical, and spectral characteristics of ECG signals were calculated and presented to a Support Vector Machine and a Multilayer Perceptron for signal quality classification. Data drawn from the CinC dataset were re-annotated by two independent annotators and another annotator for adjudication of the differences. To balance the amount of low quality and high quality data, additional noisy data samples were generated by adding noise from the PhysioNet's noise stress test database to some clean ECG signals.

In continuous hospital monitoring, false alarms increase workloads of healthcare providers, leading to decreased quality of care [18,19]. In [18], alarms in a pediatric intensive care unit were observed and it was reported that 94% of all alarms were clinically irrelevant. According to [19], only 2% to 9% of alarms were clinically relevant and important for patient treatment in intensive care units. A summary of several studies addressing the effects of false alarms in continuous monitoring was given in [20]. Different approaches to false alarm reduction in continuous cardiac monitoring have been proposed [25,26]. In [25], Aboukhalil *et al.* developed a false alarm reduction algorithm based on ECG and arterial blood pressure (ABP) signal information. Taking morphological and time information derived from ABP signals into considerations, rules were constructed for determining whether arrhythmia alarms should be suppressed. In [26], Li and Clifford described a framework for false alarm detection based on machine learning. To construct and evaluate an arrhythmia alarm classification model, features extracted from ECG, ABP, and photoplethysmogram signals were used. A genetic algorithm and a Relevance Vector Machine were employed for feature selection and classification, respectively.

This paper presents a framework for false alarm reduction in wireless continuous cardiac monitoring based on signal quality and activity type information. Signals from two sources, an ECG sensor and a 3D accelerometer, are acquired using a wireless BSN node while a subject is performing ADLs. Classification models constructed using machine learning algorithms are used for labeling ECG signals with heartbeat types and signal quality levels, and for labeling 3D acceleration signals with activity types. The obtained labels provide high-level features for a rule-based expert system to determine whether a notification of an abnormal heartbeat should be generated or ignored. Signals from three datasets were used for framework evaluation. The first dataset consists of ECG signals taken from the MIT-BIH arrhythmia database [21]. The second and third datasets consist of ECG and 3D acceleration signals acquired using BSNs from 10 young subjects and 10 elderly subjects, respectively.

This paper is organized as follows: Section 2 describes data acquisition. Section 3 describes the components of our framework. Section 4 presents the experimental settings and results. Section 5 presents a comparison with existing studies. Section 6 provides conclusions.

## 2. Data Acquisition

**Figure 1.**
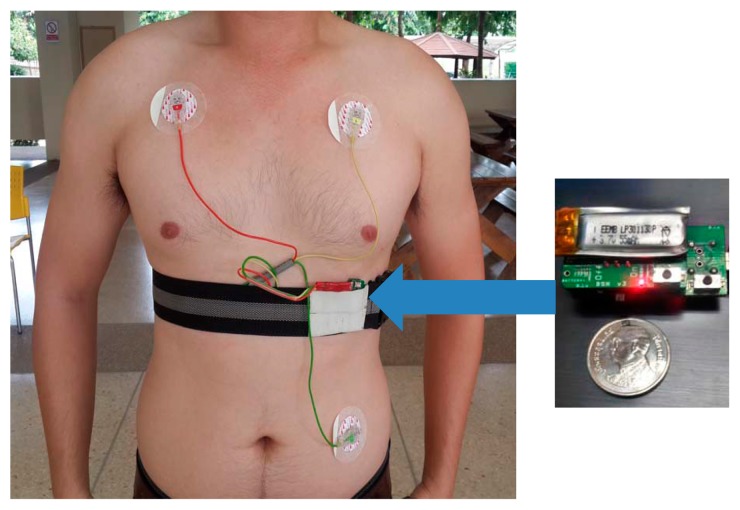
A BSN node with ECG and 3D acceleration sensors attached to a human body.

The experiments in this study involved ECG recordings from three different data sources, referred to as DS1, DS2 and DS3. The DS1 dataset was extracted from the MIT-BIH arrhythmia database [21]. It was used for evaluating arrhythmia classification and signal quality classification. The datasets DS2 and DS3 were acquired using BSN nodes [27] from 10 healthy young subjects and 10 healthy elderly subjects, respectively. These two datasets were used for evaluating arrhythmia classification, signal quality classification, activity classification, and false alarm detection. Figure 1 depicts a BSN node and the experimental setup for collecting signals in DS2 and DS3. The BSN node runs the TinyOS operating system. The hardware specification is listed in Table 1. The detailed descriptions of the three datasets are as follows.

**Figure 2.**
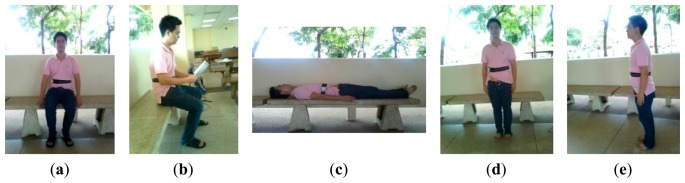
Static activities performed by young subjects (DS2): (**a**) sitting on a chair; (**b**) reading a book; (**c**) lying; (**d**) standing still; and (**e**) deep breathing.

**Figure 3.**
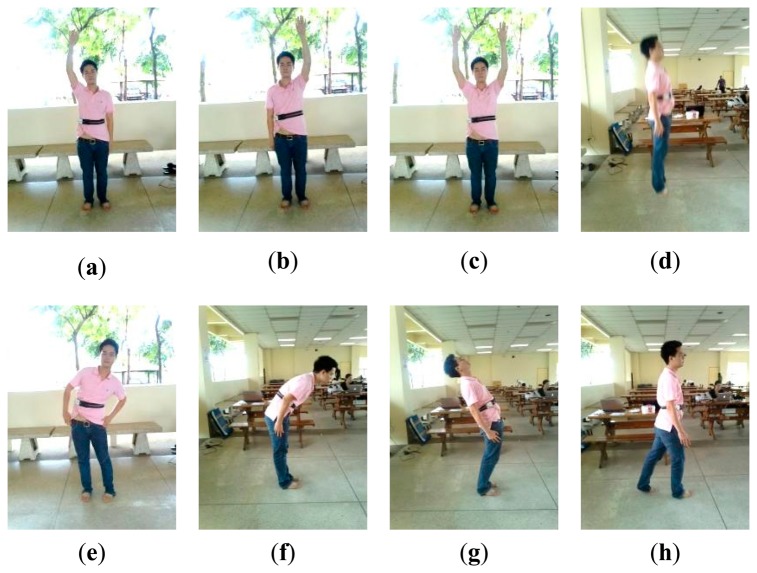

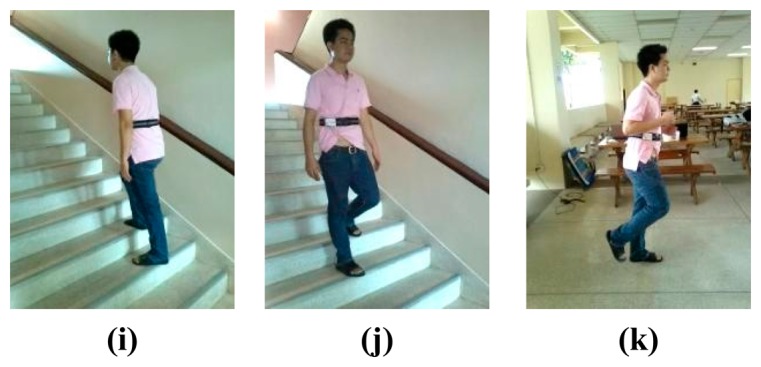
Dynamic activities performed by young subjects (DS2): (**a**) up and down movement of the right arm; (**b**) up and down movement of the left arm; (**c**) up and down movement of both arms; (**d**) jumping; (**e**) twisting left-right-left body movement at the waist; (**f**) bending forward; (**g**) bending backward; (**h**) walking; (**i**) walking up stairs; (**j**) walking down stairs; and (**k**) jogging.

**Figure 4.**
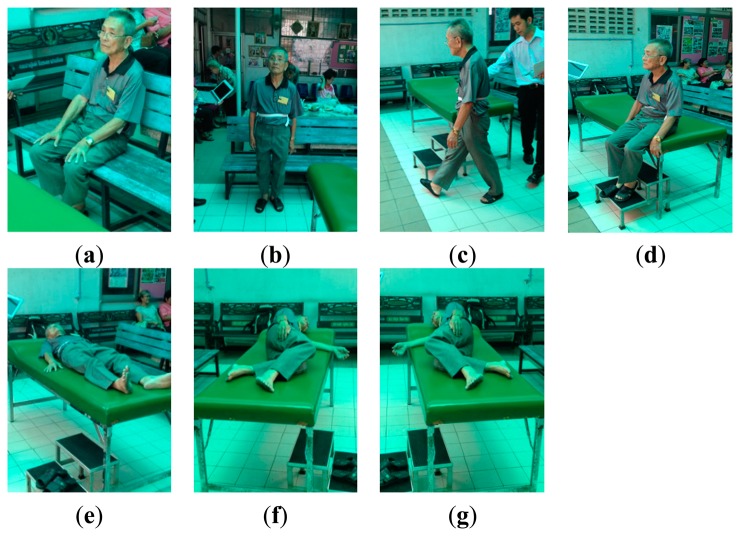
Activities performed by elderly subjects (DS3): (**a**) sitting on a chair; (**b**) standing; (**c**) walking; (**d**) sitting on a bed; (**e**) lying on the back; (**f**) lying left; and (**g**) lying right.

- *MIT-BIH arrhythmia dataset (DS1)*: The dataset DS1 consists of 48 ECG recordings, each of which is 30 min long, with a total of approximately 109,000 RR intervals. The data were acquired by the Beth Israel Hospital Arrhythmia Laboratory from 1975 to 1979. They were recorded from 25 males, aged between 32 to 89 years, and 22 females, aged between 23 to 89 years, at a sampling rate of 360 Hz using holters. Records 201 and 202 were taken from the same subject. The position and type of each beat were manually annotated by at least two independent cardiologists and the annotations were encapsulated in the dataset. Following the ANSI/AAMI EC57: 1998 standard [28], four paced recordings (102, 104, 107 and 217) were excluded.
- *A dataset acquired from healthy young subjects (DS2)*: The dataset DS2 consists of ECG (lead II configuration [29,30]) and 3D acceleration signals acquired using BSN nodes from 10 healthy subjects (seven males and three females), aged between 27 to 44 years. Each subject was monitored for approximately 30 min while performing a routine of five static activities and 11 dynamic activities, as shown in Figures 2 and 3, respectively. Each activity lasted for approximately 20 s and each subject was asked to repeat the activity routine five times.
- *A dataset acquired from healthy elderly subjects (DS3)*: The dataset DS3 consists of ECG and 3D acceleration signals acquired using BSN nodes from 10 healthy elderly subjects (two males and eight females), aged between 57 to 71 years. Based on an interview concerning his/her personal health profile, each subject had never experienced any heart disease symptom (e.g., chest pain, fainting, or severe weakness) and had never been treated for any heart problem. Each subject was monitored for approximately 5 min under a physician's supervision while performing a routine of seven activities, as shown in Figure 4.

Table 2 summarizes the characteristics of the three datasets. For QRS detection, a sampling frequency of at least 100 Hz was suggested by [31–33] in order to avoid the effects of the loss of spectral components of a QRS complex. A higher sampling rate is usually required for detecting P and T waves [31]. Taken into considerations the limitations on power consumption, computation capability, and storage resources, which are important factors for continuous monitoring using a wireless sensor, a lower sampling rate is preferred. In this study, heartbeat type classification is based solely on R-peak positions. A sampling frequency of 100 Hz was therefore used.

## 3. The Proposed Method

**Figure 5.**
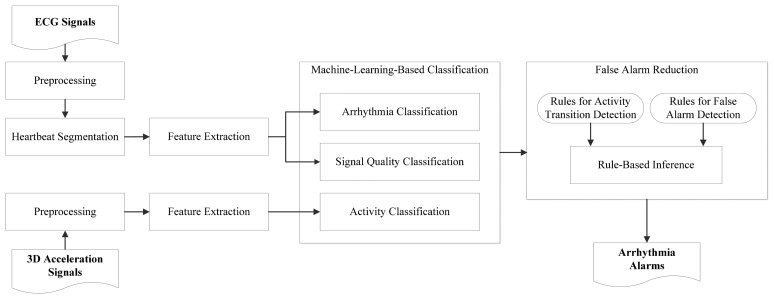
Components of the proposed framework.

Figure 5 gives an overview of our cardiac monitoring framework. Signals acquired using an ECG sensor and a 3D accelerometer are taken as input. Classification models constructed using machine learning algorithms are used for labeling input signal portions, which are then examined using a rule-based expert system. From their extracted low-level features, ECG signal portions are labeled with heartbeat types, *i.e.*, normal and abnormal heartbeats, and with signal quality levels, *i.e.*, high and low quality levels. From low-level features extracted from 3D acceleration signals, static activities are separated from non-static activities. The obtained labels, *i.e.*, heartbeat types, signal quality levels, and activity types, provide high-level features for a rule-based expert system to determine whether an arrhythmia alarms should be suppressed. For example, during a transition of activities or during a period in which signal quality is low, a predicted abnormal heartbeat should not trigger an alarm. The components of the framework are described below.

### 3.1. Preprocessing

Power line interference, electrode contact noises, motion artifacts, muscle contraction, baseline drift, and instrumentation noises generated by electronic devices [34] often cause false alarms in ECG detection. For arrhythmia classification, ECG signals were filtered using a low-pass filter for reducing noises from muscle contraction and 50-Hz power line interference. A high-pass filter was also applied to reduce noises from baseline drifts in ECG signals. For low-pass and high-pass filtering, the second-order Butterworth filters with cutoff frequencies of 15 Hz and 5 Hz [35] were used, respectively.

Z-score normalization [36] is applied to eliminate irrelevant variations due to different data sources. ECG signals in the datasets DS2 and DS3 were contaminated by noises arising from motion artifacts due to body movement while a subject performed an activity. In order to avoid the effects of such motion artifacts on the scaling parameter during normalization, outlier ECG signal samples were excluded from the calculation of the standard deviations if the difference between their amplitudes and the median was greater than 1.5 of the original standard deviations. For activity classification, Z-score normalization was also applied on the 3D acceleration signals.

### 3.2. Heartbeat Segmentation

In this study, heartbeat segmentation points, e.g., QRS onsets and R wave positions, were detected by using the “Modified So and Chan” (MSC) algorithm [37], in which a reverse R wave detection technique [38] and a digital filtering technique [39] were incorporated. This algorithm [37] was implemented in a portable ECG monitoring system and validated using ECG signals from the MIT-BIH arrhythmia database and patients in the National Taiwan University hospital (NTUH).

Using wireless ECG devices, which usually provide limited computing resources, signals can only be captured at a relatively low sampling rate. In order to deal with such devices, we modified the MSC algorithm as follows: First, only two samples are used for slope calculation, which is given by *Slope*(*t*) *= S*(*t* + 1) − *S*(*t* − 1), where *Slope*(*t*) and *S*(*t*) are the slope of ECG signals and the signal amplitude at the *t^th^* sample, respectively. Secondly, a QRS onset is detected by comparing the slope at only one sample with a slope threshold.

### 3.3. Feature Extraction

As reported in [5–7,40,41], heartbeat interval features and ECG morphology features can be used effectively for arrhythmia classification. Let R[*i*] be the R-peak position of the *i^th^* heartbeat and RR[*i*] be the time interval between R[*i*] and R[*i* − 1]. Three groups of features were calculated, based on heartbeat time intervals, ECG morphology between R-peak positions, and ECG morphology within a fixed-time interval centered at an R peak. Table 3 shows the features used for arrhythmia classification in this study.

In [42,43], statistical window-based features extracted from ECG signals for signal quality classification include mean, variance, gradient, maximum and minimum signal amplitudes, and the difference between maximum and minimum signal amplitudes. In this study, two-level features were used. Window-based features were calculated over a window of size 0.5 s, shifted by 0.25 s at each processing step. Segment-based features were then calculated on top of the window-based features over a period of 5 s.

For activity classification, deviation magnitude of the tri-axial acceleration signal calculated over a period of 5 s was used [44]. Young subjects and elderly subjects typical apply different levels of force when performing ADLs, e.g., elderly subjects usually make slower movement compared to young subjects, resulting in different levels of acceleration. In order to avoid the effects of such difference, feature values in DS3 were adjusted in proportion to the ratio between the average values of features in DS2 and DS3. The features used for signal quality and activity classifications are listed in Table 4.

### 3.4. Signal Annotation and Evaluation Measures

**Figure 6.**
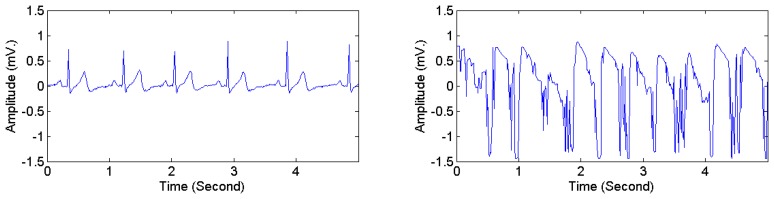
Examples of high quality ECG signals (**Left**) and low quality ECG signals (**Right**).

The heartbeat types in DS1 were divided into two categories, *i.e.*, normal and abnormal. Following the classification scheme in [28,40], four AAMI classes, *i.e.*, non-ectopic beats, supraventricular ectopic beats, fusion beats, and unknown beats (*i.e.*, beats that cannot be clearly identified using only ECG signals in lead II and time-domain-based features) were considered as normal heartbeats. Ventricular ectopic beats, including ventricular flutter or fibrillation, ventricular escape beats, and premature ventricular contraction beats, were considered as abnormal heartbeats. Table 5 presents the mapping from the original 16 heartbeat types in DS1 (originally annotated in the MIT-BIH arrhythmia dataset) to the normal type and the abnormal type. The ECG signals in DS2 and DS3 were manually annotated with signal quality levels. The signal-quality scheme recommended by [24] was adopted. Two classes were considered: high quality and low quality. The first class corresponds to the quality classes A and B in [24], *i.e.*, recordings with no visible noise and those with low-level noises that do not interfere with interpretation. The second class corresponds to the quality classes D and F in [24], *i.e.*, recordings that may be interpretable with difficulty and cannot be interpreted with confidence because of significant technical flaws. Figure 6 illustrates high quality and low quality signals in DS2. Signals in each ECG recording were divided into consecutive 5-s segments, each of which was labeled as either high or low quality. The 3D acceleration signals in DS2 and DS3 were annotated by an observer in real time with activity types and timestamps of activity transitions. The activity types were divided into static activities (e.g., sitting, lying, and standing) and non-static activities (e.g., walking and jogging).

Table 6 provides the intended meanings of positive predictions and negative predictions used in this study for arrhythmia classification, signal quality classification, and activity classification. Three statistical evaluation measures, *i.e.*, sensitivity (*Sen*), specificity (*Spec*), and accuracy (*Acc*), were employed. They are given by: *Sen* = *TP*/(*TP* + *FN*), *Spec* = *TN*/(*TN* + *FP*), and *Acc* = (*TP* + *TN*)/(*TP* + *TN* + *FP* + *FN*), where *TP*, *TN*, *FP*, and *FN* are the number of true positives, true negatives, false positives, and false negatives, respectively. With reference to Table 6, for arrhythmia classification, for example, *TP* and *TN* are the number of heartbeats correctly classified as “abnormal” and “normal”, respectively, while *FP* and *FN* are the number of those incorrectly classified as “abnormal” and “normal”, respectively.

### 3.5. Machine-Learning-Based Classification

The beat-by-beat classification scheme proposed in [40] was used for classifying heartbeats into normal heartbeats and abnormal heartbeats. Extracted features between the *i^th^* − 2 heartbeat and the *i^th^* + 1 heartbeat were used for classifying the *i^th^* heartbeat. Segment-based classification was used for predicting signal quality levels and activity types. ECG signal segments, 5 s long each, were classified into high-quality segments and low-quality segments. Likewise, either static activity or non-static activity was predicted for each 5-s 3D acceleration signal segment.

Table 7 provides a preliminary performance comparison among different classification algorithms, *i.e.*, *k*-nearest neighbor algorithm (*k*-NN), Support Vector Machine (SVM), Multilayer Perceptron (MLP), Decision Tree (C4.5), and Linear Discriminant Analysis (LDA), using 5-fold cross validation on DS1 for arrhythmia classification and using 5-fold cross validation on DS2 for signal quality and activity classification. The table shows that *k*-NN, with *k* = 3, yields the highest accuracy for arrhythmia classification and is comparable with the other algorithms for signal quality and activity classification. The *k*-NN algorithm is a non-parametric instance-based learning algorithm and is among the simplest machine learning algorithms [36]. It was employed in several related studies for various purposes, e.g., arrhythmia classification [8,45,46], signal quality classification [47], and activity classification [48,49]. This algorithm was also selected in this study for constructing classification models since it gives relatively high classification accuracy, is easy to implement, and yields more consistent classification results across different implementations. For *k*-NN, only the value of *k* and the distance metric (in our study, standard Euclidean distance) are needed to be defined, whereas some other algorithms may yield different classification results depending on the quantization method in use and/or initial model parameters. However, it should be noted that our framework is not restricted to classification using *k*-NN. Other classification algorithms can also be employed. The main focus of the framework is the use of classification results obtained from machine-learning-based algorithms in combination with rules for false alarm reduction.

### 3.6. False Alarm Reduction

For constructing a rule base for false alarm reduction using ECG and 3D acceleration signals, domain-specific knowledge concerning the effects of ADLs on ECG signals and characteristics of activities under examination is used. In this study, the following domain-specific knowledge is considered:

- During an activity transition [16], ECG signals are often corrupted by motion artifacts and should therefore be excluded.
- During dynamic activities [17], ECG signals are often contaminated with noises and signal quality should be taken into considerations.
- When examining non-static activities information about previous activities and post activities can be used to distinguish activity transitions from dynamic activities.
- The heart rate of a middle-aged normal subject ranges from 60 to 160 beats per minute while the subject is taking exercise [50]. The average heart rate of a normal subject during static activities ranges from 60 to 100 beats per minute.

**Figure 7.**
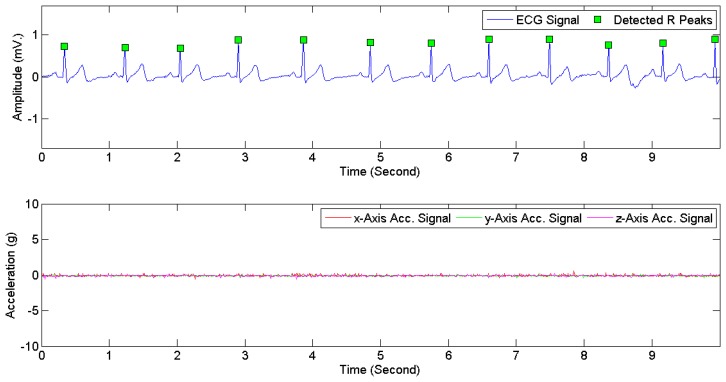
ECG signals (**Above**) and 3D acceleration signals (**Below**) when a subject was sitting.

**Figure 8.**
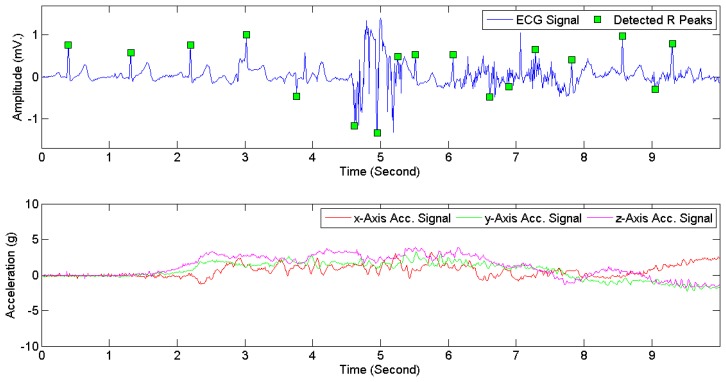
ECG signals (**Above**) and 3D acceleration signals (**Below**) when a subject made a transition from lying to standing.

**Figure 9.**
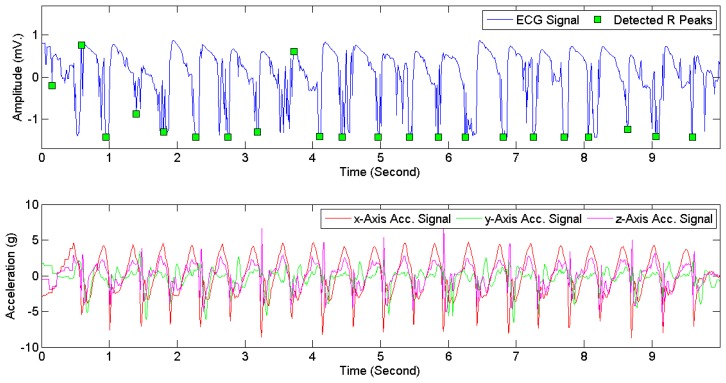
ECG signals (**Above**) and 3D acceleration signals (**Below**) when a subject was jogging.

Figure 7 provides examples of ECG signals and 3D acceleration signals while a subject was sitting. Figures 8 and 9 illustrate noises arising from motion artifacts in ECG signals during a transition from lying to standing and during a subject was jogging, respectively.

**Figure 10.**
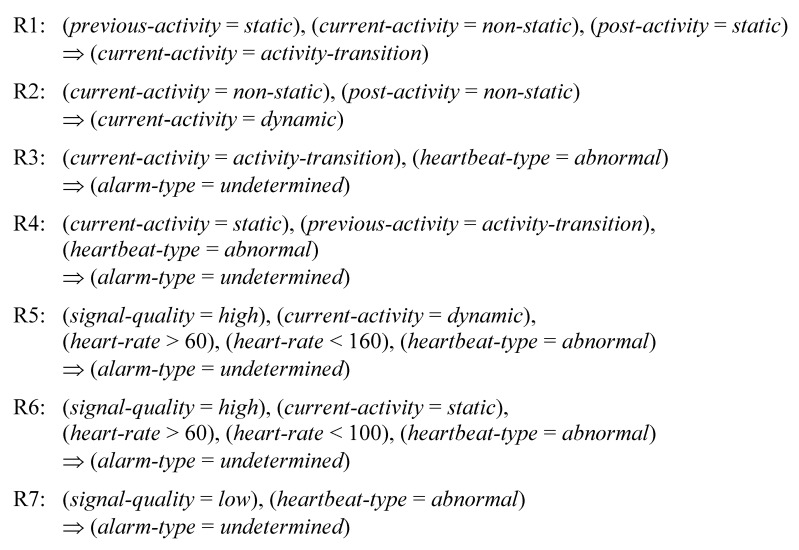
A rule set for detecting false alarms.

Seven rules, as listed in Figure 10, were constructed for false alarm detection. The terms “normal” heartbeat type, “abnormal” heartbeat type, “high” signal quality, and “low” signal quality are described in Section 3.4. The term “static” activity refers to sitting, reading while sitting, lying (on the back, left, right), standing still, or deep breathing while standing still. “Non-static” activities include activity transitions and dynamic activities such as walking, jogging, and jumping.

The rules R1 and R2 are used for dividing non-static activities into activity transitions and dynamic activities. For example, the rule R1 states that a non-static activity should be classified as an activity transition when its previous and post activities are both detected as static activities.

The rules R3–R7 are used for verifying arrhythmia alarms. The rule R3 excludes arrhythmia alarms occurring during activity transitions. Since an activity transition often results in continuing slight electrode movements, the rule R4 disables an alarm occurring in a static-activity segment that immediately follows a transition. The rule R5 excludes an arrhythmia alarm generated from high quality signals during a dynamic activity if the extracted heart rate is within the heart rate range of a normal subject while taking exercise. Using the rule R6, an arrhythmia alarm generated from high quality signals is ignored if the predicted activity type is static and the extracted heart rate is within the normal range. The rule R7 excludes arrhythmia alarms generated from signals with low quality.

## 4. Experiments and Results

### 4.1. Experiment Settings

Classification models were developed in Java using the IBk algorithm (with *k* = 3) through the Weka API [36]. Table 8 summarizes the dataset usage for arrhythmia classification, signal quality classification, and activity classification in our experiments, along with model validation schemes. The details are as follows:

- *Arrhythmia classification*: Based on the arrhythmia evaluation method proposed in [5–7,9], DS1 was divided into two datasets, *i.e.*, a training set (DS1_A_) and a test set (DS1_B_), each of which contains ECG signals from 22 recordings with almost the same distribution of heartbeats from each arrhythmia types. Recordings in DS1_A_ and DS1_B_ are given in Table 9. To investigate the performance of arrhythmia classification on ECG signals acquired from wireless sensors while subjects are performing ADLs, the entire datasets DS2 and DS3 were also used as test sets.
- *Signal quality classification*: Leave-one-out cross validation [36] was applied to ECG signals in DS2, *i.e.*, signal recordings from nine subjects were used for training and those from one subject was used for testing. The process was repeated ten times in order that the signal recordings from each subject were used once for testing. To evaluate signal quality classification on an independent dataset, the model constructed from the entire DS2 was tested against DS3. To investigate the robustness of the signal quality model on ECG signals with cardiac arrhythmias, the model was also evaluated on DS1.
- *Activity classification*: The 3D acceleration signals obtained from DS2 were used for evaluating the performance of activity classification by using leave-one-out cross validation. The model constructed from the entire DS2 was tested against DS3. Activity classification is not evaluated on DS1 since no acceleration signal is recorded in this dataset.

### 4.2. Results

For arrhythmia classification, the evaluation results are shown in Table 10. The obtained accuracy, sensitivity, and specificity on DS1_B_ (49,587 beats) were 97.80%, 86.66%, and 98.57%, respectively. The accuracy values on DS2 (18,702 beats) were 94.75% and 88.81% for static and non-static activities, respectively, and those on DS3 (3168 beats) were 88.53% and 88.75% for static and non-static activities, respectively. Since ECG signals in DS2 and DS3 were acquired from healthy subjects, all heartbeats in these two datasets were annotated as normal beats. Accordingly, *TP* and *FN* were both zero for DS2 and DS3. However, due to noises and artifacts arising from ADLs, some beats in these two datasets were incorrectly classified as abnormal, *i.e.*, *FP* was not zero.

Table 11 demonstrates the performance of signal quality classification. The accuracy obtained from leave-one-out cross-validation on DS2 (2506 segments) was 94.81%. When the signal quality model constructed from DS2 was evaluated against DS1 (15,884 segments) and DS3 (469 segments), the accuracy values were 99.07% on DS1 and 90.41% on DS3. A closer examination on DS1 reveals that 148 segments (from a total of 15,884 segments) were classified as low signal quality. These segments altogether consist of 1273 heartbeats (1.26% of all heartbeats in DS1). They are divided into 1069 normal heartbeats and 204 abnormal heartbeats. Most of these abnormal heartbeats are Premature Ventricular Contractions (PVCs). Note that the total number of PVCs in DS1 is 6903 heartbeats and 97.04% of them (6699 heartbeats) were classified as high quality.

Table 12 presents the performance of activity classification. Using leave-one-out cross-validation, the obtained accuracy, sensitivity, and specificity on DS2 (2506 segments) were 88.15%, 89.43%, and 86.40%, respectively. The activity classification model constructed from DS2 was tested against DS3 (469 segments). The obtained accuracy, sensitivity, and specificity were 86.35%, 97.47%, and 84.10%, respectively.

In order to investigate the effects of ECG signal quality using BSN-based cardiac monitoring while the subjects are performing ADLs, the rules for false alarm detection were evaluated on the datasets DS2 and DS3. With reference to Table 10, Table 13 compares the classification results before and after the false arrhythmia alarms detected by the rules were excluded. The exclusion raised the accuracy values from 90.73% to 98.69% on DS2 and from 88.57% to 97.89% on DS3.

The overall accuracy increased from 90.42% to 98.57%, *i.e.*, the overall false alarm rate reduced from 9.58% to 1.43%. The experimental results show that signal quality and ADLs information can be used for enhancing the performance of cardiac continuous monitoring using wireless sensors.

## 5. Related Works

A summary of related works is provided in Table 14. All of these studies mainly focus on signal quality classification and false alarm reduction. For signal quality classification, Hayn *et al.* [23] presented 4 ECG signal quality indices (SQIs), *i.e.*, empty lead criterion, spike detection criterion, lead crossing point criterion, and a measure of QRS detection robustness. Applying rules constructed from a combined use of the four indices on datasets extracted from the CinC dataset [22], the average accuracy of 92.5% was achieved. Clifford *et al.* [24] proposed an algorithm for signal quality classification using 7 SQIs, which were presented in their previous work [51]. From twelve-lead ECG signals in the CinC dataset [22], 84 features based on these SQIs were extracted. Several combinations of SQIs for signal quality classification were evaluated. Using Support Vector Machine (SVM), an accuracy of 94.9% was obtained by the resulting best combination. In [42], Schumm *et al.* described a framework for signal quality assessment in ambulatory ECG monitoring. Using a contactless ECG system installed into a backrest of an airplane seat, ECG signals were captured from 12 subjects while they were performing five ADLs, *i.e.*, entertainment, working, reading, sleeping, and eating. Quality labels were defined as good or bad based on a comparison between R-peak positions extracted from two differrent ECG sources, *i.e.*, their contactess ECG sensor and a ground truth ECG device (a commercial device). Using Logistic Regression (LR) for signal quality classification, an accuracy of 92.0% was reported. None of [23,24,42] investigated false alarms in continous cardiac monitoring.

Most works on false alarm reduction in continuous cardiac monitoring employed the PhysioNet's MIMIC II database for framework evaluation, e.g., [25,26,52,53]. The MIMIC II database [54] is a large multi-parameter intensive care unit database consisting of ECG signals, arterial blood pressure (ABP) signals, photoplethysmogram (PPG) signals, central venous pressure (CVP) signals, and pulmonary arterial pressure (PAP) signals. In [25], Aboukhalil *et al.* used rules derived from ABP signal information for verifying whether arrhythmia alarms should be accepted. Five alarm categories were considered, *i.e.*, Asystole (ASYS), Extreme Bradycardia (EB), Extreme Tachycardia (ET), Ventricular Tachycardia (VT), and Ventricular Fibrillation (VF). Using their proposed rules, false alarms were reportedly reduced from 42.7% to 17.2%. Li and Clifford [26] applied a Relevance Vector Machine (RVM) to construct an arrhythmia alarm classification model using information extracted from ECG, ABP, and PPG signals. Using a genetic algorithm for feature selection, false EB alarms were reduced from 26.6% to 1.3%. In [52], Sayadi and Shamsollahi developed a nonlinear joint dynamical state-space model based on a combination of ECG, ABP, PPG, CVP, and PAP signal information. Using Bayesian filters as classifiers, false alarms for the ASYS, EB, ET, VT, and VF categories were reduced from 42.3% to 9.9%. In [53], Salas-Boni *et al.* described an algorithm for detecting false VT alarms based solely on ECG signals. Using a L_1_-regularized Logistic Regression (L_1_-LR) classifier, with features based on Multi-resolution Wavelet Transform, false VT alarms were reduced from 73.0% to 54.4%. The works reported in [25,26,52,53] aimed to reduce the number of false ECG-based alarms in hospital environments. None of these studies has taken acceleration signals into considerations nor reported signal quality classification results.

An important challenge for continuous ECG monitoring is signal quality inconsistency due to motion artifacts induced by different physical activities. This issue has been addressed in a few recent studies. In [55], Hu *et al.* reported that ECG signals were unreliable during walking and suggested that a human activity should be considered as an observation in their proposed layered hidden Markov model. The study, however, only reported arrhythmia classification results on 16 ECG recordings selected from the MIT-BIH arrhythmia database, and did not demonstrate the actual use of acceleration signals in the arrhythmia classification experiment. In [56], Takalokastari *et al.* reported a significant correlation between noises in ECG signals and levels of acceleration signals based on an analysis of ECG and 3D acceleration signals acquired from 30 subjects during 3 types of ADLs, *i.e.*, running, biking, and walking. However, the use of signal quality information and ADL types for reducing false arrhythmia alarms was not investigated. To the best of our knowledge, this paper presents the first study on BSN-based arrhythmia monitoring that demonstrates a complete arrhythmia detection process by using signal quality and activity information to improve the detection accuracy, with validation on data collected from real subjects using a BSN device.

## 6. Conclusions

A framework for reducing arrhythmia false alarms in continuous cardiac monitoring using wireless BSNs has been presented, based on ECG and 3D acceleration signals. In this study, we focused on investigating the effects of signal quality and noises arising from body movements during ADLs on arrhythmia alarms. Machine-learning-based classifiers were used for classifying heartbeat types and signal quality levels from features extracted from ECG signals, and also for classifying activity types from features extracted from 3D acceleration signals. With signal quality and activity type information, a rule-based expert system was used for determining whether abnormal heartbeats should be ignored. For framework evaluation, signals from three different sources were employed, *i.e.*, the MIT-BIH arrhythmia database, a dataset acquired using BSNs from 10 young subjects, and a dataset acquired using BSNs from 10 elderly subjects. The evaluation showed the overall reduction of false alarms from 9.58% to 1.43%. The study demonstrated the potential use of acceleration signals for false alarm reduction and for development of home cardiac monitoring.

## Figures and Tables

**Table 1.** Specification of the BSN nodes used in this study.

**Module**	**Parameter**	**Specification**
Processor (TI MSP430F1611)	Flash memory	48 KB
	RAM	10 KB
	On-chip ADC resolution	12 bit
	ADC channels	8 channels
	DAC channels	2 channels

Radio transceiver (TI CC2420)	Wireless communication standard	IEEE 802.15.4 (2.4 GHz)
	Data rate	250 Kbps
	Ranges indoor and outdoor	50 m and 125 m

EEPROM (AT 45DB321)	Flash memory	4 MB
	SRAM buffers	512/528 bytes
	Program/Erase cycle	100,000 cycles

**Table 2.** A summary of dataset descriptions.

**Characteristics**	**DS1**	**DS2**	**DS3**
Device type	Hospital-based holter	Wireless BSN	Wireless BSN
Signal type	ECG	ECG and 3D acceleration	ECG and 3D acceleration
November of subjects	47 subjects	10 subjects	10 subjects
Age	23–89 years	27–44 years	57–71 years
Sampling rate	360 Hz	100 Hz	100 Hz
Activity type	N/A	16 ADLs	7 ADLs

**Table 3.** R-peak-based features used for arrhythmia classification.

**Group Label**	**Features**
Heartbeat interval features	RR[*i*]
	Variance of {RR[*i*], RR[*i* + 1}
	Variance of {RR[*i* − 1], RR[*i*], RR[*i* + 1}

ECG morphology between R-peak positions	Mean of signal amplitudes between R[*i* − 1] and R[*i*]
	Absolute difference between signal amplitudes at R[*i* − 1] and R[*i*]
	Absolute difference between signal amplitudes at R[*i*] and R[*i* + 1]

ECG morphology within a fixed-time interval centered at an R peak	Mean of signal amplitudes within a 0.12-s interval
	Mean of signal amplitudes within a 0.16-s interval
	Mean of gradients of signal amplitudes within a 0.12-s interval
	Mean of gradients of signal amplitudes within a 0.16-s interval
	Mean of gradients of signal amplitudes within a 0.23-s interval
	Variance of gradients of signal amplitudes within a 0.06-s interval
	Difference between the maximum and minimum signal amplitudes within a 0.08-s interval

**Table 4.** Segment-based features used for signal quality classification and activity classification.

**Group Label**	**Features**
ECG statistical features (for signal quality classification)	Mean of signal amplitude means
	Mean of signal amplitude variances
	Variance of signal amplitude variances
	Gradient of signal amplitude variances
	Minimum of signal amplitude means
	Minimum of signal amplitude variances
	Difference between the maximum and minimum signal amplitude variances
	Difference between the maximum and minimum variances of absolute signal amplitudes
	Mean of means of absolute signal amplitudes

Acceleration signal features (for activity classification)	Deviation magnitude

**Table 5.** Heartbeat types associated with beats in DS1.

**AAMI Class**	**MIT-BIH annotation**	**Description**	**Classification**
Non-ectopic beat	N	Normal beat	Normal (93,486 beats)
	L	Left bundle branch block beat	
	F	Right bundle branch block beat	
	j	Nodal (junctional) escape beat	
	e	Atrial escape beat	

Supraventricular ectopic beat	a	Aberrated atrial premature beat	
	S	Ectopic supraventricular beat	
	A	Atrial premature contraction	
	J	Nodal (junctional) premature beat	

Fusion beat	F	Fusion of ventricular and normal beat	

Unknown beat	/	Paced beat
	Q	Unclassifiable beat
	F	Fusion of paced and normal beat

Ventricular ectopic beat	!	Ventricular flutter/fibrillation	Abnormal (7470 beats)
	E	Ventricular escape beat	
	V	Premature ventricular contraction	

**Table 6.** The meanings of positive predictions and negative predictions.

**Classification**	**Prediction**	

	**Positive**	**Negative**
Arrhythmia	Abnormal heartbeat	Normal heartbeat
Signal quality	Low signal quality	High signal quality
Activity	Non-static activity	Static activity

**Table 7.** Performance comparison of classification algorithms (using 5-fold cross validation on DS1 for arrhythmia classification and 5-fold cross validation on DS2 for signal quality and activity classification).

**Algorithm**	**Classification**								

	**Arrhythmia**			**Signal Quality**			**Activity**		

	***Sen***	***Spec***	***Acc***	***Sen***	***Spec***	***Acc***	***Sen***	***Spec***	***Acc***
*k*-NN	95.2%	99.7%	99.4%	79.7%	98.3%	96.0%	87.0%	88.9%	88.1%
SVM	89.3%	99.7%	98.9%	83.6%	97.5%	95.8%	89.4%	90.9%	90.3%
MLP	93.1%	99.4%	98.9%	78.8%	99.0%	96.5%	89.4%	91.1%	90.4%
C4.5	94.1%	99.6%	99.2%	77.5%	99.1%	96.4%	87.3%	91.8%	89.9%
LDA	82.0%	95.6%	94.6%	78.1%	98.4%	95.8%	82.9%	93.6%	89.1%

**Table 8.** Dataset usage for classification evaluation.

**Dataset Usage**	**Classification**		

	**Arrhythmia**	**Signal Quality**	**Activity**
Training set	DS1_A_	DS2	DS2
Test set	DS1_B_, DS2, DS3	DS1, DS3	DS3
Leave-one-out evaluation	None	DS2	DS2

**Table 9.** Separating training and test datasets in DS1.

**Dataset**	**November of Beats**	**MIT-BIH Arrhythmia Recordings**
DS1_A_ (Training set)	51,369	101, 106, 108, 109, 112, 114, 115, 116, 118, 119, 122, 124, 201, 203, 205, 207, 208, 209, 215, 220, 223, 230

DS1_B_ (Test set)	49,587	100, 103, 105, 111, 113, 117, 121, 123, 200, 202, 210, 212, 213, 214, 219, 221, 222, 228, 231, 232, 233, 234

**Table 10.** Evaluation results: Arrhythmia classification.

**Dataset**	**Activity Type**	***TP***	***TN***	***FP***	***FN***	***Acc***	***Sen***	***Spec***
DS1	Static	2787	45,710	661	429	97.80%	86.66%	98.57%

DS2	Static	0	5736	318	0	94.75%	N/A	94.75%
	Non-static	0	11,233	1415	0	88.81%	N/A	88.81%
	All	0	16,969	1733	0	90.73%	N/A	90.73%

DS3	Static	0	2238	290	0	88.53%	N/A	88.53%
	Non-static	0	568	72	0	88.75%	N/A	88.75%
	All	0	2806	362	0	88.57%	N/A	88.57%

**Table 11.** Evaluation results: Signal quality classification.

**Dataset**	**Activity Type**	***TP***	***TN***	***FP***	***FN***	***Acc***	***Sen***	***Spec***
DS1	Static	0	15,736	148	0	99.07%	N/A	99.07%

DS2	Static	44	885	8	16	97.48%	73.33%	99.10%
	Non-static	183	1264	38	68	93.17%	72.91%	97.08%
	All	227	2149	46	84	94.81%	72.99%	97.90%

DS3	Static	52	293	34	7	89.38%	88.14%	89.60%
	Non-static	12	67	2	2	95.18%	85.71%	97.10%
	All	64	360	36	9	90.41%	87.67%	90.91%

**Table 12.** Evaluation results: Activity classification.

**Dataset**	***TP***	***TN***	***FP***	***FN***	***Acc***	***Sen***	***Spec***
DS2	915	1294	153	144	88.15%	89.43%	86.40%
DS3	77	328	62	2	86.35%	97.47%	84.10%

**Table 13.** Arrhythmia classification results after false alarm reduction on DS2 and DS3.

**Dataset**	**Activity Type**	**Without False Alarm Reduction**			**With False Alarm Reduction**		

		**Normal**	**Abnormal**	***Acc***	**Normal**	**Abnormal**	***Acc***
DS2	Static	5736	318	94.75%	5996	58	99.04%
	Non-static	11,233	1415	88.81%	12,461	187	98.52%
	All	16,969	1733	90.73%	18,457	245	98.69%

DS3	Static	2238	290	88.53%	2483	45	98.22%
	Non-static	568	72	88.75%	618	22	96.56%
	All	2806	362	88.57%	3101	67	97.89%

Overall		19,775	2095	90.42%	21,558	312	98.57%

**Table 14.** Comparison with related studies.

**Ref.**	**Dataset**	**Device Type**	**Classifier**	**Signals Used**	**Accuracy of Signal Quality Classification**	**Study Purpose**
[23]	CinC2011	Mobile phone	Rules	ECG	92.5%	Signal quality classification
[24]	CinC2011	Mobile phone	SVM	ECG	94.9%	Signal quality classification
[42]	ECG recordings while subjects were performing 5 ADLs	Contactless ECG system	LR	ECG	92.0%	Signal quality classification
[25]	MIMIC II	Hospital-based station	Rules	ECG + ABP	N/A	False alarm reduction in an ICU setting
[26]	MIMIC II	Hospital-based station	RVM	ECG + ABP + PPG	N/A	False alarm reduction in an ICU setting
[52]	MIMIC II	Hospital-based station	Bayesian	ECG + ABP + PPG + CVP + PAP	N/A	False alarm reduction in an ICU setting
[53]	MIMIC II	Hospital-based station	L_1_-LR	ECG	N/A	False alarm reduction in an ICU setting
Our work	ECG recordings while subjects were performing 16 ADLs	Wireless BSN	*k*-NN + Rules	ECG + 3D acceleration	92.6%	False alarm reduction in a free living environment
